# Neurologic Symptoms and Cerebrovascular Events During Atogepant Therapy: A Case Series with Contextual Comparison with a Non-Gepant–Treated Migraine Cohort

**DOI:** 10.3390/jcm15051930

**Published:** 2026-03-03

**Authors:** Carl H. Göbel, Axel Heinze, Katja Heinze-Kuhn, Anna Cirkel, Hartmut Göbel

**Affiliations:** 1 Kiel Migraine and Headache Center, 24149 Kiel, Germany; heinze@schmerzklinik.de (A.H.); khk@schmerzklinik.de (K.H.-K.); anna.cirkel@uni-luebeck.de (A.C.); hg@schmerzklinik.de (H.G.); 2 Department of Neurology, University Hospital Schleswig-Holstein, Campus Kiel, 24149 Kiel, Germany; 3 Department of Neurology, University Hospital Schleswig-Holstein, Campus Lübeck, 23562 Lübeck, Germany

**Keywords:** atogepant, CGRP receptor antagonists, posterior circulation, ischemia, microvascular dysfunction, neurovascular resilience, migraine prevention, gepants, case series

## Abstract

**Background:** CGRP contributes to cerebrovascular regulation, mainly based on experimental and translational data; human evidence remains limited. Gepants, including atogepant, are effective migraine preventives and achieve partial penetration across the blood–brain barrier. However, their neurologic and cerebrovascular safety in heterogeneous patient populations remains incompletely characterized. **Objective:** To describe acute neurologic events observed during atogepant therapy, provide contextual information regarding their baseline occurrence, and explore potential mechanisms by which CGRP receptor blockade may influence neurovascular resilience. **Methods:** We report five adults treated with atogepant (30–60 mg/day) who developed acute neurologic symptoms prompting emergency hospital admission. All patients underwent comprehensive diagnostic assessment including neuroimaging, vascular studies, cardiac evaluation, and laboratory testing. To provide context, a retrospective comparison cohort of migraine patients not treated with gepants during a similar period was analyzed. Baseline characteristics were summarized, and event occurrence was compared using Fisher’s exact test. **Results:** Among 575 individuals treated with atogepant, five experienced acute neurologic events, including one cerebellar infarction and several transient focal syndromes without structural correlates. No cerebrovascular events requiring hospitalization were identified in the non-gepant cohort (*n* = 610). In an unadjusted analysis, this difference was statistically significant (*p* = 0.027). The events were clinically heterogeneous, and several lacked radiologic confirmation of ischemia. Conventional vascular risk factors were present in some patients. **Conclusions:** These findings do not imply causality but raise the possibility that CGRP receptor blockade may reduce cerebrovascular adaptability in susceptible individuals. Clinicians should remain vigilant for ischemia or microvascular dysfunction when patients receiving atogepant present with acute vertigo, diplopia, ptosis, or hemisensory symptoms—even when CT and CTA are normal—and obtain timely MRI and vascular assessment. The absence of comparable events in a retrospective non-gepant cohort provides contextual information but does not permit inference regarding increased risk due to potential confounding and unmeasured factors. The findings are exploratory and hypothesis-generating, underscoring the need for prospective controlled studies to clarify the cerebrovascular safety of CGRP receptor antagonists in routine clinical practice.

## 1. Introduction

CGRP contributes to nociceptive transmission, trigeminovascular activation, and cerebral vasodilation, mainly based on experimental and translational data; human evidence remains limited [1,2,3]. Beyond its role in migraine biology, CGRP exerts protective effects in ischemic conditions by maintaining microvascular perfusion, stabilizing endothelial integrity, and modulating neuroinflammation [4,5]. Pharmacologic inhibition of CGRP signaling, whether through monoclonal antibodies or gepants, may therefore influence neurovascular adaptability, particularly in individuals with preexisting small-vessel disease or reduced cerebrovascular reserve [6,7,8,9,10,11].

Gepants have demonstrated favorable tolerability in clinical trials [12,13,14,15], yet these studies included relatively few patients with cerebrovascular comorbidities, and their ability to partially cross the blood–brain barrier introduces theoretical concerns regarding central autoregulatory pathways [11,16,17,18,19,20,21]. Real-world safety data in broader populations remain limited.

We report five patients (Table 1) who developed neurologic symptoms—including sensory deficits, cranial neuropathies, vertigo, ataxia, and cerebellar infarctions—during ongoing atogepant therapy. These cases illustrate a spectrum of neurovascular phenomena observed under CGRP receptor blockade and underscore the need for systematic evaluation of gepant safety in individuals with vascular risk factors.

## 2. Methods

### 2.1. Study Design and Setting

This retrospective case series was conducted at a tertiary headache center and describes patients who developed acute neurologic symptoms during ongoing preventive treatment with atogepant. The observation period corresponded to routine clinical practice without a predefined study protocol.

The number of individuals treated with atogepant during the observation period was obtained from aggregate prescribing records. Individual-level data on vascular risk factors, cumulative treatment duration, and person-time exposure were not systematically available for the entire treated population, precluding formal estimation of incidence rates or adjusted risk comparisons.

### 2.2. Case Identification and Clinical Assessment

Patients were identified through clinical records based on the occurrence of acute neurologic symptoms during atogepant therapy that prompted medical evaluation. All cases underwent comprehensive diagnostic work-up according to clinical standards, including neurological examination, brain imaging (computed tomography and/or magnetic resonance imaging), vascular studies, cardiac assessment, and laboratory testing as clinically indicated.

Clinical data collected for each case included demographic characteristics, migraine history, atogepant dosage and treatment duration, symptom characteristics, diagnostic findings, management, and clinical outcomes.

### 2.3. Comparison Cohort

To provide contextual information regarding the baseline occurrence of cerebrovascular events, a retrospective comparison cohort of migraine patients treated during a similar time period who did not receive gepants was identified from the same clinical setting. Patients could receive other preventive therapies, including monoclonal antibodies targeting the CGRP pathway, reflecting routine clinical practice.

Baseline demographic characteristics were summarized for descriptive comparison. Systematic data on vascular risk factors and comorbidities were not consistently available for the entire cohort; therefore, no adjustment for potential confounders was possible. The comparison analysis should be interpreted as exploratory and descriptive rather than as a formal assessment of relative risk or causal association. The observation period for both cohorts was approximately 12 months.

### 2.4. Outcome Definition

The primary outcome was the occurrence of acute neurologic events requiring hospital evaluation during the observation period. Events were classified based on clinical presentation and diagnostic findings, including radiologically confirmed infarction and transient neurologic syndromes without structural correlates.

### 2.5. Statistical Analysis

Continuous variables are reported as mean ± standard deviation. Categorical variables are presented as counts and percentages. Between-group comparisons for categorical variables were performed using Fisher’s exact test. Continuous variables were compared using Student’s *t*-test. All statistical tests were two-sided, and a *p*-value < 0.05 was considered statistically significant. Given the retrospective design, incomplete covariate data, and low event frequency, the analyses were unadjusted and intended to provide contextual descriptive information rather than inferential estimates of treatment effect. Statistical analyses were performed using IBM SPSS Statistics, Version 30 (IBM Corp., Armonk, NY, USA).

## 3. Results

### 3.1. Case 1

A 48-year-old woman with a long-standing history of migraine with and without aura presented with the sudden onset of severe vertigo lasting approximately one hour. She experienced difficulty walking with lateral deviation. She had been receiving atogepant 60 mg daily for several months, with a substantial reduction in monthly migraine days and improved functional capacity. On the day of symptom onset, she noted an abrupt spinning sensation when rising from bed, followed by unsteadiness when walking. She did not experience headache, diplopia, dysarthria, limb weakness, or sensory changes. On examination, she was fully alert and oriented, with a normal cranial nerve assessment and no motor or sensory deficits. Blood pressure and cardiovascular examination were normal. Computed tomography (CT) and computed tomography angiography (CTA) of the head and neck revealed no hemorrhage, stenosis, or large-vessel occlusion. Due to persistent symptoms, MRI was obtained and demonstrated a new acute infarction in the left posterior inferior cerebellar artery (PICA) territory, confirming a posterior circulation stroke despite normal vascular imaging. Duplex ultrasonography showed no stenosis, and transesophageal echocardiography (TEE) excluded cardioembolism or patent foramen ovale (PFO). Coagulation studies and inflammatory markers were normal.

Because no alternative etiology was identified, the stroke was classified as cryptogenic. She was started on aspirin and statin therapy. Symptoms improved steadily over the following week. The temporal association with atogepant raised the question of impaired microvascular compensation as a possible contributor.

### 3.2. Case 2

A 53-year-old woman with migraine without aura began treatment with atogepant 30 mg daily approximately one month before presentation. She reported early improvement in headache frequency but developed sudden binocular diplopia while working at a computer. Within minutes, she noted tingling and mild weakness of the left hand and difficulty focusing on objects at varying distances. She denied headache, vertigo, dysarthria, or additional limb symptoms.

Neurologic examination revealed intact visual acuity but impaired convergence with subtle difficulty maintaining binocular alignment. Sensory testing demonstrated paresthesia in the left hand and slight weakness of the left arm with a pronator drift. Gait and coordination were normal. CT and CTA showed no hemorrhage, stenosis, or dissection. Magnetic resonance imaging (MRI) of the brain, obtained shortly after symptom onset, revealed no diffusion restriction or structural abnormalities. Laboratory studies, including electrolytes, inflammatory markers, glucose, and coagulation parameters, were within normal limits. Echocardiography demonstrated normal systolic function and no embolic source.

Her symptoms resolved completely within 24 h. The differential diagnosis included vertebrobasilar transient ischemic attack (TIA), migraine aura with atypical sensory features, and transient ocular motor dysfunction. The close temporal relationship to the initiation of atogepant raised the possibility of reduced posterior circulation adaptability during vascular stress. She continued preventive therapy with monitoring.

### 3.3. Case 3

A 52-year-old man with migraine with and without aura was enrolled in an open-label study of atogepant 60 mg daily, with a reduction in headache frequency from approximately 12 monthly headache days to fewer than three. After several months of stable preventive therapy, he developed sudden unilateral hypoesthesia involving the left cheek, jaw, neck, arm, and anteromedial thigh. He described the sensation as “reduced texture and temperature perception,” accompanied by intermittent burning in the left periorbital region. Symptoms began following an emotionally stressful interpersonal confrontation but persisted independently of emotional triggers. He reported no headache or typical aura symptoms.

Examination showed mild left ptosis (pre-existing), new facial asymmetry with slight drooping of the left side of the mouth, and patchy hypoesthesia in the left face and leg. Motor strength, reflexes, gait, and coordination were normal. CT revealed no acute lesion. MRI including diffusion-weighted imaging (DWI) and fluid-attenuated inversion recovery (FLAIR) sequences showed no ischemia, demyelination, mass lesion, or brainstem abnormality. Duplex ultrasound demonstrated mild non-stenosing macroangiopathy without flow disturbance and no sonographic signs of arterial dissection or intramural hematoma. Somatosensory evoked potentials (SSEPs) of the tibial and median nerves showed minimally delayed but symmetric latencies, arguing against a focal conduction defect. Long-term cardiac monitoring revealed no arrhythmia.

Symptoms remained stable at follow-up without radiologic correlates. Although functional mechanisms and migraine-related sensory disturbance were considered, the unilateral distribution and cranial autonomic feature (ptosis) suggested possible transient microvascular dysfunction. Atogepant therapy was discontinued.

### 3.4. Case 4

A 62-year-old woman with chronic migraine with aura and typical aura without headache, treated with atogepant 60 mg daily for three weeks, presented with a three-day history of persistent facial weakness characterized by left-sided ptosis, left facial droop, and periorbital hypoesthesia. She had developed a typical migraine headache the following day, which had already resolved, while the facial weakness persisted.

Neurologic examination showed clear left-sided ptosis, reduced periorbital sensation, and mild facial asymmetry with drooping of the left side of the mouth. Coordination, strength, and reflexes were intact. CT was normal. MRI demonstrated no acute infarction, hemorrhage, mass lesion, or cranial nerve enhancement. Chronic white-matter hyperintensities were present, consistent with migraine-associated or age-related small-vessel disease. Vascular ultrasonography showed no stenosis, and cardiac evaluation revealed no pathology.

Although she had never previously experienced ptosis as part of her aura, a diagnosis of persistent migraine aura was deemed most consistent. Symptoms resolved gradually over the following weeks.

### 3.5. Case 5

A 70-year-old man with migraine with and without aura, hypertension, hyperlipidemia, and a remote transient ischemic attack (TIA) six years earlier was treated with atogepant 60 mg daily for four days. He developed abrupt paresthesia in the right hand without a headache.

On examination, aside from the right-hand paresthesia, no abnormalities were found. Six years earlier, he had been diagnosed with a TIA presenting with right arm weakness and numbness lasting 24 h. CT showed no acute lesion. MRI revealed chronic infarctions in the right PICA and superior cerebellar artery (SUCA) territories, with no new diffusion restriction. CTA and duplex ultrasound showed no stenosis or occlusion. Transthoracic echocardiography demonstrated normal ejection fraction and no thrombus or valvular disease.

Given the abrupt onset and known posterior circulation vulnerability, a TIA was considered likely. His statin therapy was intensified. Because atogepant was the only recent pharmacologic change and no alternative mechanism was identified, reduced microvascular compensation during CGRP receptor blockade was discussed as a possible contributing factor, and treatment was discontinued.

### 3.6. Comparison Cohort Analysis

A comparison cohort of 610 migraine patients not treated with gepants during a similar observation period was identified. Patients could receive other preventive therapies, including monoclonal antibodies targeting the CGRP pathway, reflecting routine clinical practice. Demographic characteristics were broadly comparable between groups (Table 2).

No cerebrovascular events requiring hospitalization were observed in the non-gepant cohort, whereas five events occurred in the atogepant-treated cohort (0.9%). In an unadjusted Fisher’s exact test, this difference reached statistical significance (*p* = 0.027).

## 4. Discussion

This case series highlights recurring clinical patterns that raise the possibility that neurologic symptoms may emerge during atogepant therapy in individuals with underlying neurovascular vulnerability. Across five patients among 575 individuals treated with atogepant during the observation period, neurologic symptoms emerged during ongoing therapy and prompted emergency hospital admission, including cerebellar infarctions as well as transient hemisensory or cranial nerve disturbances without structural correlates (Table 1). Although several presentations involved posterior circulation territories, others—such as hemifacial hypoesthesia or periorbital sensory loss—were not confined to a single vascular distribution, indicating a broader range of neurovascular involvement. This heterogeneity suggests that CGRP receptor blockade may influence both central and peripheral neurovascular mechanisms [1,2,10]. Symptom onset ranged from 4 days to several months after treatment initiation, arguing against a uniform early exposure effect. Acute management included standard stroke evaluation, antiplatelet therapy when appropriate, and individualized decisions regarding continuation or discontinuation of atogepant. The clinical presentations were heterogeneous, and only one case demonstrated radiologically confirmed infarction, whereas others lacked structural correlates and remain diagnostically uncertain.

A limitation of this case series is the absence of systematic individual-level vascular risk data for the entire treated cohort of 575 patients. As a result, direct comparisons of demographic and vascular risk profiles between patients with neurologic events and the remaining atogepant-treated population were not feasible. The retrospective design, lack of systematic vascular risk factor data, and small number of events limit interpretation and preclude estimation of incidence or relative risk. Available human studies using transcranial Doppler and perfusion imaging have not demonstrated clinically relevant impairment of cerebral hemodynamics under CGRP pathway inhibition, suggesting preserved global autoregulation in most individuals [22]. Our observations may therefore reflect vulnerability in selected individuals rather than a general drug effect. Future prospective registries with structured vascular phenotyping will be required to quantify relative risk and identify susceptible subgroups. To provide contextual information regarding baseline event occurrence, a retrospective comparison cohort of migraine patients treated during a similar period without gepant therapy was analyzed. Some patients in this cohort received monoclonal antibodies targeting the CGRP pathway, reflecting real-world clinical practice. No cerebrovascular events requiring hospitalization were identified in that cohort, whereas five events occurred in the atogepant-treated population. This unadjusted comparison should be interpreted cautiously because systematic vascular risk factor data were not available and causal inference cannot be drawn. Nevertheless, detailed phenotyping of the five cases suggests that advanced age, migraine with aura, and preexisting vascular vulnerability may represent relevant modifiers of neurovascular resilience.

**Figure 1:**
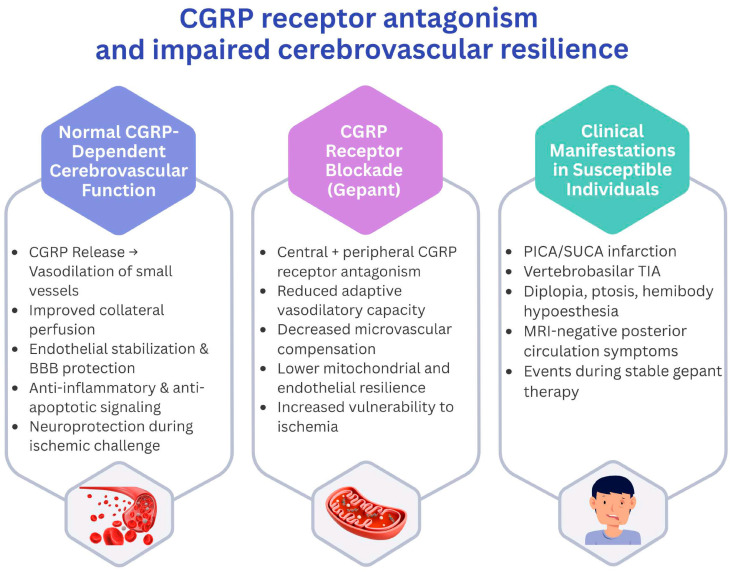
Conceptual model illustrating how CGRP receptor antagonism might influence cerebrovascular resilience, particularly within the posterior circulation. Under physiologic conditions, CGRP contributes to vasodilation of small-caliber intracranial vessels, stabilization of endothelial and mitochondrial function, and maintenance of collateral perfusion. Gepants, including atogepant, partially penetrate the blood–brain barrier and antagonize central and peripheral CGRP receptors. During ischemic challenge—especially in territories with limited collateral capacity such as PICA and SUCA—CGRP blockade could theoretically reduce adaptive vasodilation, weaken microvascular compensation, attenuate neuroprotective signaling and thereby potentially increase clinical vulnerability to transient or overt ischemic symptoms.

CGRP is thought to contribute to cerebrovascular homeostasis and adaptive vasodilation, largely supported by experimental and translational evidence [1,3,8,10,16,23]. It supports adaptive vasodilation, maintains endothelial stability, and contributes to mitochondrial protection and anti-inflammatory signaling under ischemic stress (Figure 1). Experimental models demonstrate that loss of CGRP signaling—whether through genetic deficiency or receptor blockade—impairs cerebrovascular autoregulation and increases infarct size during hypoperfusion [24]. Individuals with preexisting microangiopathy or limited collateral reserve may therefore be especially sensitive to perturbations in this pathway [25,26,27]. However, these experimental findings may not directly translate to clinical settings, and available human studies have generally not demonstrated clinically meaningful impairment of cerebral hemodynamics under CGRP pathway inhibition.

Posterior circulation structures are classically more vulnerable to ischemia due to their limited collateralization [28,29]. While several events in this series were consistent with this pattern, others occurred outside the vertebrobasilar system. Importantly, diffusion-weighted imaging may yield false-negative results in early posterior circulation ischemia [30]. Moreover, stroke associated with migraine with aura often presents as cryptogenic and with fewer conventional vascular risk factors. Therefore, the absence of radiologic correlates in several cases does not fully exclude transient ischemic mechanisms [31]. Transient sensory and motor disturbances may reflect localized dysfunction of small vessels or neurovascular junctions beyond posterior territories. These findings align with the pharmacologic properties of gepants, which—as small-molecule receptor antagonists—exhibit partial penetration across the blood–brain barrier and therefore have the potential to influence CGRP-dependent regulatory circuits in the brainstem, cerebellum, trigeminal system, and periorbital sensory pathways [32,33,34]. Alternative explanations, including migraine aura variants or functional neurologic symptoms, cannot be excluded in cases without objective imaging findings. Accordingly, the present report describes neurologic events temporally associated with atogepant therapy rather than events proven to be caused by the medication.

Pharmacologic distinctions between gepants and monoclonal antibodies targeting the CGRP pathway may also be relevant. Gepants directly block the CGRP receptor and achieve variable daily receptor occupancy, in contrast to the more stable peripheral ligand sequestration produced by monoclonal antibodies [3,8,12,33,34,35]. Because CGRP is a potent vasodilator that supports microvascular perfusion under metabolic or ischemic challenge, receptor-level antagonism may theoretically diminish neurovascular compensation during periods of physiological stress [36,37]. In addition, CGRP contributes to autonomic and sensorimotor integration within central brainstem circuits; partial central receptor blockade could therefore provide a plausible explanation for transient posterior circulation-like symptoms without corresponding structural abnormalities [34,38]. These considerations remain theoretical and are not supported by direct clinical evidence from the present series.

These observations do not imply causality, but they highlight a biologically plausible association warranting systematic investigation. The internal consistency of the cases—symptom onset during active therapy, lack of alternative etiologies, and alignment with known cerebrovascular physiology—supports the hypothesis that CGRP receptor blockade may reduce microvascular adaptability in susceptible individuals [36]. Older age, microangiopathy, and prior cerebrovascular events may further diminish compensatory reserve, increasing vulnerability to transient or overt ischemic phenomena during physiological stress [24,37,39].

Clinically, the findings underscore the need for vigilance when evaluating patients receiving atogepant who present with acute vertigo, diplopia, ptosis, or hemisensory disturbances. Even when initial imaging is reassuring, timely MRI and vascular evaluation remain essential. Therapeutic decisions should be individualized, balancing documented benefit in migraine prevention against the possibility of reduced cerebrovascular resilience in high-risk individuals. Ultimately, prospective studies enriched for patients with vascular comorbidities are needed to clarify incidence, mechanisms, and clinical relevance. As gepants are increasingly used across diverse patient populations, defining their cerebrovascular safety profile has become an important area for future research.

## Figures and Tables

**Table 1:** Demographics, migraine history, and temporal association between atogepant exposure and neurologic events in five patients. The table outlines the demographics, migraine history and diagnosis/diagnoses, current and former treatment, atogepant dose and vascular risk factors. The final columns describe the nature of the events and diagnostic findings. All events manifested during ongoing atogepant therapy.

	Case 1	Case 2	Case 3	Case 4	Case 5
Demographics	48-year-old female	53-year-old female	52-year-old male	62-year-old female	70-year-old male
Migraine history	36 years	29 years	26 years	48 years	16 years
Migraine diagnosis/diagnoses	Migraine without aura Migraine with aura 8 days/month pre-Atogepant 5 days/month with Atogepant	Migraine without aura 10 days/month pre-Atogepant 4 days/month with Atogepant	Migraine without aura Migraine with aura 12 days/month pre-Atogepant 1–2 days/month with Atogepant	Chronic migraine with aura Typical aura without headache 15 days/month pre-Atogepant	Migraine without aura Migraine with aura 12 days/month pre-Atogepant
Acute treatment	Zolmitriptan 5 mg oral Zolmitriptan 5 mg nasal Dimenhydrinat 50 mg oral	Sumatriptan 100 mg oral	Rizatriptan 10 mg Eletriptan 40 mg	Metamizole 1000 mg	Lasmiditan 100 mg Paracetamol 500 mg + Codeine 30 mg
Atogepant dose and duration before event	Atogepant 60 mg for 6 months	Atogepant 30 mg for 7 weeks	Atogepant 60 mg for 6 months	Atogepant 60 mg for 3 weeks	Atogepant 60 mg for 4 days
Other prophylactic treatment	Opipramol 50 mg	Topiramate 50 mg	none	None	None
Vascular risk factors	Smoking: no Hypertension: no Hypercholesterinemia: no Obesity: no Estrogen therapy: no Cardiovascular or cerebrovascular events: no	Smoking: no Hypertension: no Hypercholesterinemia: no Obesity: no Estrogen therapy: yes Cardiovascular or cerebrovascular events: no	Smoking: no Hypertension: yes Hypercholesterinemia: no Obesity: yes Cardiovascular or cerebrovascular events: no	Smoking: no Hypertension: no Hypercholesterinemia: yes Obesity: no Estrogen therapy: no Cardiovascular or cerebrovascular events: no	Smoking: no Hypertension: yes Hypercholesterinemia: yes Obesity: no Cardiovascular or cerebrovascular events: TIA 6 years ago
Nature of neurologic event	Vertigo lasting 1 h Unsteady, laterally deviating gait for one week	Diplopia, weakness left hand, paresthesia left hand lasting 24 h	Hypoesthesia and Hemiparesthesia left side, facial weakness with drooping of left oral commissure Ongoing > 2 months	Facial weakness with ptosis and drooping of left oral commissure, periorbital hypoesthesia	Paresthesia right hand for 5 h
Stroke-MRI	Cerebellar infarction in the PICA territory	No infarction	No infarction	No infarction white-matter lesions, consistent with migraine-related or age-associated small-vessel disease	No new infarction Chronic cerebellar infarctions in the PICA and SUCA-territory right side
pFO	no	no	no TEE or TTE	no	no

**Table 2:** Baseline characteristics and cerebrovascular events in patients treated with atogepant and a non-gepant comparison cohort. Data are presented as mean ± standard deviation or number (percentage), as appropriate. *p*-values were calculated using Fisher’s exact test for categorical variables and Student’s *t*-test for continuous variables. Owing to the lack of systematic vascular risk factor data, the comparison is unadjusted and exploratory. The comparison cohort included patients receiving standard migraine preventive therapies other than gepants, including monoclonal antibodies targeting the CGRP pathway. Migraine subtypes were not mutually exclusive; some patients experienced both migraine with and without aura.

Characteristic	Atogepant Cohort	Control Cohort	*p*-Value
Number of patients (N)	575	610	
Age, years (mean ± SD)	49.02 ± 13.69	47.65 ± 14.30	*p* = 0.091
Sex			
male, *n* (%)	76 (13.2%)	92 (15.1%)	*p* = 0.361
female, *n* (%)	499 (86.8%)	518 (84.9%)	
Migraine without Aura			
yes	564 (98.1%)	593 (97.2%)	*p* = 0.345
no	11 (1.9%)	17 (2.8%)	
Migraine with Aura			
yes	267 (46.4%)	300 (49.2%)	*p* = 0.352
no	308 (53.6%)	310 (50.8%)	
Cerebrovascular events			
yes	5 (0.9%)	0 (0%)	*p* = 0.027
no	575 (99.1%)	610 (100%)	

## Data Availability

Anonymized clinical data supporting the findings of this case series are available from the corresponding author upon reasonable request.

## Supplementary Materials

The following supporting information can be downloaded at: https://www.mdpi.com/article/10.3390/jcm15051930/s1, File S1: CARE checklist.

## Glossary

BBB	Blood–Brain Barrier
CGRP	Calcitonin Gene–Related Peptide
CTA	Computed Tomography Angiography
CT	Computed Tomography
DWI	Diffusion-Weighted Imaging
FLAIR	Fluid-Attenuated Inversion Recovery (MRI sequence)
MHDs	Monthly Headache Days
MRI	Magnetic Resonance Imaging
PICA	Posterior Inferior Cerebellar Artery
PFO	Patent Foramen Ovale
SSEP	Somatosensory Evoked Potentials
SUCA	Superior Cerebellar Artery
TEE	Transesophageal Echocardiography
TIA	Transient Ischemic Attack
