# Supplementary material for: Neurologic Symptoms and Cerebrovascular Events During Atogepant Therapy: A Case Series with Contextual Comparison with a Non-Gepant–Treated Migraine Cohort
Source: J Clin Med. 2026 Mar 3;15(5):1930. doi: 10.3390/jcm15051930 (PMC12985913; doi:10.3390/jcm15051930)
Supplement: Supplementary file 1 [file jcm-15-01930-s001.zip › jcm-4179861-supplementary.pdf]

CARE Checklist
Neurologic symptoms and cerebrovascular events in patients treated with atogepant: a case series
Göbel et al.

1. Title
CARE requirement: Identify as case report or case series and include the key phenomenon.
Fulfilled in manuscript: Yes — title explicitly states 'a case series' and mentions the neurologic events associated with atogepant.

2. Keywords
CARE requirement: 2–5 key terms.
Fulfilled: Yes — migraine, atogepant, CGRP receptor blockade, posterior circulation, adverse neurologic events.

3. Abstract
CARE requirement: Structured summary with background, clinical findings, interventions, outcomes.
Fulfilled: Yes — Structured abstract with Background, Objective, Methods, Results, Conclusions.

4. Introduction
Requirement: Scientific and clinical background; rationale for reporting the case(s).
Fulfilled: Yes — describes CGRP physiology, gepant relevance, and rationale for reporting posterior circulation events.

5. Patient Information
Requirement: Demographics, medical history, previous interventions, relevant lifestyle/clinical factors.
Fulfilled: Yes — each case includes age, sex, migraine subtype, vascular risk factors, prior medical history.

6. Presentation (Clinical Findings)
Requirement: Symptoms, examination findings.
Fulfilled: Yes — detailed description of onset, neurologic status, clinical examination.

7. Timeline
Requirement: Chronological summary of symptoms, diagnostics, treatments, and outcomes.
Fulfilled: Yes — timelines incorporated into narrative; Table 3 represents temporal association; cases presented in chronological clinical sequence.

8. Diagnostic Assessment
Requirement: Diagnostic methods; challenges; rationale for conclusions; differential diagnoses.
Fulfilled: Yes — CT/CTA, MRI, duplex ultrasound, cardiac evaluation, labs; differential diagnoses addressed (migraine aura, functional disorder, TIA, ischemia).

9. Diagnostic Challenges
Requirement: Uncertainty, normal imaging despite symptoms, atypical presentation.
Fulfilled: Yes — posterior circulation ischemia with initially normal imaging explicitly discussed; MRI-negative ischemia addressed.

10. Therapeutic Interventions
Requirement: Medical, surgical, preventive, pharmacologic interventions with doses and duration.
Fulfilled: Yes — all patients were on atogepant with specific dose (30–60 mg) and treatment duration before symptom onset; secondary prevention documented when relevant.

11. Follow-Up and Outcomes
Requirement: Clinical course, changes after intervention, objective and subjective outcomes.
Fulfilled: Yes — each case describes evolution, neurologic outcome, symptom resolution or persistence.

12. Discussion
Requirement: Interpretation, literature context, strengths, limitations, implications.
Fulfilled: Yes — extensive discussion of CGRP physiology, differences between gepants and mAbs, posterior circulation vulnerability, clinical implications, need for future research.

13. Patient Perspective (optional)
Requirement: Optional short commentary by patient.
Fulfilled: No — omitted intentionally, appropriate for case series and not required by Neurology.

14. Informed Consent
Requirement: Documentation of consent for publication.
Fulfilled: Yes — included in manuscript ("All patients provided informed consent…").

15. Adverse Event Extension (CARE AE Extension)
Because this case series concerns **possible drug-associated neurologic events**, the AE extension applies.
AE-1: Temporal Relationship
Fulfilled: Table 3 and narrative explicitly detail timing between atogepant exposure and symptom onset.
AE-2: Causality Considerations
Fulfilled: Discussion covers biological plausibility, absence of alternative etiologies, and mechanistic rationale.
AE-3: Dechallenge/Rechallenge (if applicable)
Fulfilled: No rechallenge occurred; dechallenge not a main diagnostic feature. Stated appropriately.
AE-4: Exclusion of Other Causes

Fulfilled: Comprehensive diagnostic workup detailed (imaging, labs, cardiac evaluation, Doppler studies).

AE-5: Seriousness, Reversibility, Outcome

Fulfilled: Outcomes stated clearly; events ranged from transient deficits to confirmed infarction.

16. Completeness Statement

All CARE and CARE Adverse Event Extension items applicable to this case series have been addressed in the manuscript.
